# Leadless Left Bundle Branch Area Pacing in Cardiac Resynchronisation Therapy: Advances, Challenges and Future Directions

**DOI:** 10.3389/fphys.2022.898866

**Published:** 2022-06-06

**Authors:** Nadeev Wijesuriya, Mark K. Elliott, Vishal Mehta, Baldeep S. Sidhu, Marina Strocchi, Jonathan M. Behar, Steven Niederer, Christopher A. Rinaldi

**Affiliations:** ^1^ School of Biomedical Engineering and Imaging Sciences, King’s College London, London, United Kingdom; ^2^ Department of Cardiology, Guy’s and St Thomas’ NHS Foundation Trust, London, United Kingdom

**Keywords:** leadless cardiac resynchronization therapy, heart failure, cardiac resynchoronization therapy, leadless cardiac pacing, endocardial pacing, conduction system pacing, left bundle branch area pacing

## Abstract

Leadless left bundle branch area pacing (LBBAP) represents the merger of two rapidly progressing areas in the field of cardiac resynchronisation therapy (CRT). It combines the attractive concepts of pacing the native conduction system to allow more physiological activation of the myocardium than conventional biventricular pacing, with the potential added benefits of avoiding long-term complications associated with transvenous leads via leadless left ventricular endocardial pacing. This perspective article will first review the evidence for the efficacy of leadless pacing in CRT. We then summarise the procedural steps and pilot data for leadless LBBAP, followed by a discussion of the safety and efficacy of this novel technique. Finally, we will examine how further mechanistic evidence may shed light to which patients may benefit most from leadless LBBAP, and how improvements in current experience and technology could promote widespread uptake and expand current clinical indications.

## Introduction

Cardiac Resynchronisation Therapy (CRT) is a well-established treatment for symptomatic dyssynchronous heart failure (Glikson et al., 2021). Conventional CRT involves transvenous lead-based systems which provide biventricular (BiV) pacing from leads in the right ventricle (RV), and in the coronary sinus to achieve epicardial left ventricular (LV) stimulation. CRT delivered in a conventional approach significantly improves symptoms whilst reducing hospitalisations and mortality in indicated patients (McAlister et al., 2007).

However, despite widespread uptake and success, studies consistently demonstrate that over 30% of implanted patients fail to derive benefit from conventional CRT (Young, 2003). Several factors contribute to this limited efficacy. The implant location of the LV lead is restricted to the epicardial regions encompassed by the patient’s coronary venous system (Wouters et al., 2021), which may cause difficulty in targeting optimal sites and avoiding transmural LV scar. LV scar is present in up to 40% of CRT candidates, and predicts poor response (Bleeker et al., 2006; Chalil et al., 2007; Leyva et al., 2011; Wong et al., 2013). In addition, modelling studies demonstrate that conventional CRT does not replicate physiological stimulation across the endocardium and in some instances may be pro-arrhythmic (Mendonca Costa et al., 2019). Furthermore, over 4% all CRT candidates are unable to receive lead-based CRT systems due to: unfavourable coronary venous anatomy preventing initial LV lead implant; poor upper limb venous access; or a prohibitively high infection risk (Gamble et al., 2016). Novel technologies, including leadless LV endocardial pacing and conduction system pacing (CSP) have emerged to effectively treat these challenging patient cohorts. Both therapies have the potential to overcome the anatomical and physiological drawbacks of conventional epicardial CRT.

Several studies have reported favourable acute outcomes with LV endocardial pacing. (Garrigue et al., 2001; Derval et al., 2010; Ginks et al., 2011; Padeletti et al., 2012; Morgan et al., 2016). Endocardial stimulation provides more physiological LV electrical activation compared to epicardial pacing. (Bordachar et al., 2012). Importantly, endocardial pacing provides the benefit of unrestricted LV pacing locations, which can be vital in patients with unfavourable characteristics such as myocardial scar. However, initial lead-based systems to deliver LV endocardial pacing were accompanied by a prohibitively high risk of stroke (Morgan et al., 2016) and a need for lifelong anticoagulation.

Leadless LV endocardial pacing offers several advantages over lead-based systems. Complete device endothelialisation subverts the stroke risk and anticoagulation requirement. (Echt et al., 2010; Okabe et al., 2022). These devices can be implanted in patients where venous access or infection issues prohibit both conventional and lead-based endocardial CRT (Gamble et al., 2016). In addition, leadless pacing can overcome the numerous long-term complications associated with transvenous leads including: fracture (1–4%); tricuspid regurgitation (5%); venous obstruction (8–21%); and infection (1–2%) (Bernard, 2016) which often result in the need for high risk lead extraction procedures. The WiSE-CRT system (EBR Systems Inc., Sunnyvale CA) is a leadless LV pacing system which is commercially available in the European Economic Area, and has been granted Breakthrough Device Designation by the US Food and Drug Administration (FDA) (Auricchio et al., 2014). The system consists of a battery connected to an ultrasound transmitter, which is implanted subcutaneously at the fourth, fifth or sixth intercostal place, and the receiver electrode, which is implanted in the LV cavity via aortic or trans-septal access (Supplementary Appendix Figure S1). The system requires the patient to have a “co-implant” *in-situ* capable of producing continuous right ventricular (RV) pacing, which can be either a conventional device, such as a pacemaker or implantable cardioverter defibrillator (ICD), or a leadless RV pacing device such as MICRA^TM^ (Medtronic, Minneapolis MN). The transmitter and battery detect an RV pacing pulse emitted by the co-implant. Within 10 ms of detection of the RV pacing stimulus, the transmitter emits a number of ultrasound pulses to locate the receiver electrode. Once the transmitter is electronically optimally aligned, a longer ultrasound wave is emitted, which is detected and converted to a pacing stimulus by the receiver electrode. This results in LV pacing, and thereby BiV pacing. Observational (Auricchio et al., 2014; Reddy et al., 2017; Carabelli et al., 2021; Okabe et al., 2022) and registry studies (Sieniewicz et al., 2020) have demonstrated the effectiveness of leadless endocardial pacing in complex real-world patients with poor cardiac function and multiple co-morbidities, and the international multicentre SOLVE-CRT trial is ongoing (Okabe et al., 2022).

One area of ongoing research is identifying the optimal site for leadless LV endocardial pacing. Unlike conventional CRT, leadless technology allows pacing from any location within the LV cavity. There can be substantial individual variation reported in optimal endocardial pacing sites (Sohal et al., 2014; Behar et al., 2016). due to factors such as scar and underlying conduction disease. Studies have investigated whether pre-procedural imaging or intra-procedural electroanatomical mapping can be used to guide the optimal LV pacing location for leadless CRT (Sieniewicz et al., 2018a; Sieniewicz et al., 2018b; Sidhu et al., 2020). These guidance techniques were all developed on the basis of targeting the areas of latest LV myocardial activation, which tended to be in the posterior or lateral walls. Interestingly however, Salden et al. (2020) reported that temporary LV endocardial septal pacing consistently gave superior improvement in LV activation times (LVAT) compared to other endocardial locations and postulated that this was through capture of the native conduction system rather than septal myocardium alone. It is therefore possible that traditional parameters which guide optimal LV pacing location such as scar and late activation, may not apply in the context of CSP.

In this perspective article, we first describe the procedure for leadless CSP. We then examine the safety and efficacy of this novel technology. Finally, we discuss the limitations, unanswered questions, and potential future directions of leadless CSP in the context of all available CRT modalities.

## Feasibility of Left Ventricular Endocardial Septal Pacing

Histological studies have demonstrated that the left sided His-Purkinje system runs in close proximity to the LV septal endocardial surface (Padala et al., 2021). The majority of patients display three branches: a long left anterior fascicle, a shorter posterior fascicle, and an intermediate branch which forms a fan-like structure. Electroanatomical mapping (EAM) studies have validated histological findings in both structurally normal hearts (Long et al., 2013), and in patients with left bundle branch block (LBBB) (Upadhyay et al., 2019), who comprise the majority of the CRT population. The extensive nature of the left bundle branch network is postulated to be the reason why lead-based left bundle branch area pacing (LBBAP) (Huang et al., 2019) results in larger R waves and lower thresholds than His-Bundle pacing (HBP) (Hua et al., 2020), as the network forms a much larger target area than the penetrating His Bundle for lead deployment and electrical capture.

Data from these EAM studies have also suggested that CSP is able to effectively correct LBBB (Upadhyay et al., 2019). performed EAM in 72 patients with LBBB to identify the pattern of conduction tissue disease. They reported that in 64% of patients, the conduction disease phenotype was that of proximal His-Purkinje block. The remaining patients displayed intact Purkinje activation, with electrical dyssynchrony presumably caused by intramural conduction delay. HBP corrected the QRS duration in 85% of patients with proximal conduction disease block, and in 0% of patients with intact Purkinje activation. They hypothesized that CSP was therefore likely to correct electrical dyssynchrony in the majority of patients with LBBB. There has been significant uptake of CSP, with transvenous LBBAP in particular garnering interest in view of high reported success rates from observational studies (Padala and Ellenbogen, 2020). In the last year, there have been significant developments in the field of leadless LV endocardial pacing which has seen the emergence of leadless LBBAP.

Whilst theoretically the WiSE-CRT receiver electrode can be implanted anywhere within the LV cavity, until recently, all implants were targeted to the basal lateral/posterior walls (Auricchio et al., 2014; Reddy et al., 2017; Sieniewicz et al., 2020). In these initial observational studies, the vast majority of implants were performed using a retrograde aortic approach via femoral artery access. With this approach and the unidirectional delivery sheath, stable targets can only be feasibly achieved on the lateral LV walls, thus limiting implant locations. It has only been with the recent emergence of a trans-septal technique (Sieniewicz et al., 2017) for receiver electrode deployment that the operator is able to target the LV septal wall, thus performing LBBAP with the aim of capturing the native conduction system.

To date, there has been one published case report of LV septal pacing (Elliott et al., 2021). Elliott et al. report the case of a patient with ischaemic cardiomyopathy, with an existing dual chamber pacemaker implanted via a persistent left superior vena cava. A tunnelled LV lead was placed via the right subclavian vein (RSV) which subsequently failed. After failure of the original RV lead, a new one was placed via the RSV. A new LV lead implant was also attempted, but was unsuccessful due to coronary sinus thrombosis. A WiSE-CRT procedure was therefore performed. No suitable thresholds for endocardial electrode implantation were found on the LV lateral wall. The decision was therefore made to implant septally (Supplementary Appendix Figure S2).

The procedure for LV endocardial septal pacing involves mapping the LV septum using a decapolar catheter, in order to identify a left bundle potential (LBP). In the presence of LBBB or complete heart block, high output temporary HBP from the right side may generate a LBP. If this fails, RV pacing can be used to map a retrograde LBP. A steerable sheath, such as the Medtronic FlexCath Advance^TM^ is then used to target the receiver electrode to the desired location where it is deployed. Whilst this case report demonstrated the feasibility of leadless LBBAP, (Elliott et al., 2021), key questions are yet to be addressed prior to expansion of this technique with regards to safety and efficacy.

## Can Leadless LBBAP be Performed Safely?

WiSE-CRT implantation is an invasive procedure which is performed in a co-morbid population with very few alternative treatment options, and carries an increased risk profile compared to standard CRT implantation. A systematic review and meta-analysis of studies to date (Wijesuriya et al., 2022) reported a procedure/device related complication rate of 23.8% with a procedure related mortality of 2.8%, and a procedure success rate of 91%. It should be noted that early feasibility lead-based CRT studies reported a similar complication rates ranging from 23 to 28% (van Rees et al., 2011). Following initially high rates of pericardial effusion in the WiSE-CRT study, (Auricchio et al., 2014), the delivery mechanism was updated, reducing the risk of tamponade to 1.2%. Whilst still significant, this rate is comparable to other commonly-performed invasive procedures such as radiofrequency ablation (Bollmann et al., 2018). Improved operator experience will likely also improve the overall risk profile. In the WiSE Registry (Sieniewicz et al., 2020), 76% of complications occurred within a centre’s first 10 cases, suggesting a learning curve. The most recent data released from the roll-in phase of the ongoing SOLVE-CRT (Okabe et al., 2022) trial showed a procedure related complication rate of 9.7% with no mortalities, which would suggest that updated techniques and technologies have improved the procedural safety profile.

With regards to safety, an area which is particularly pertinent to leadless LBBAP is the transition from a retrograde aortic technique to a trans-septal technique. A meta-analysis of 181 patients revealed a femoral arterial vascular access complication rate of 5% (Wijesuriya et al., 2022). The trans-septal technique is likely to result in a considerably lower rate of vascular complications compared to the retrograde aortic technique, especially as the operators in this field of cardiology tend to have significantly more experience in trans-septal procedures.

## Assessing the Potential Efficacy of Leadless LBBAP

The efficacy of leadless CRT using the WiSE-CRT system has been reported in several observational studies (Auricchio et al., 2014; Reddy et al., 2017; Sieniewicz et al., 2020; Carabelli et al., 2021; Okabe et al., 2022). A meta-analysis of these studies reports a pooled echocardiographic response rate [increase in LV ejection fraction (LVEF) of >5%] of 54%, and a mean increase in LVEF of 6.3%. Patients in these studies represented a difficult to treat population, with 22% being non-responders to standard CRT (Wijesuriya et al., 2022). Notably, all patients in these studies received LV lateral wall electrode implants. Evidence for the efficacy of LV endocardial LBBAP is, to date, from in-silico computer simulation studies, case series, small observational studies and mechanistic cohort studies.

The technique for leadless LV septal endocardial pacing targeting the LBBA was first described by Elliott et al. (Elliott et al., 2021) The authors report a case where temporary pacing at the site of the LBP showed greater electrical resynchronization (QRS duration 106 ms) compared to pacing at the mid-lateral LV wall (QRS 132 ms) and baseline RV pacing (172 ms). In addition, LBBAP resulted in an equivalent acute haemodynamic response compared to BiV pacing from various lateral wall endocardial locations. This was consistent with an earlier observation study by Salden et al. (2020), where temporary LV septal endocardial pacing was performed in 27 patients undergoing CRT implantation, and acute improvement measured through QRSd, QRS area using vectorcardiography, standard deviation of activation times (SDAT) using multi-electrode body surface mapping, and haemodynamic response (LV dP/dt). They reported that LV septal pacing resulted in a larger reduction in QRS area (to 73 ± 22 μVs) and SDAT (to 26 ± 7 ms) than BiV pacing (to 93 ± 26 μVs and 31±7 ms; both *p* < 0.05).

Whilst leadless septal pacing targeting a LBP has been performed using the WiSE-CRT system (Elliott et al., 2021), whether this results in CSP is yet to be determined. The WISE-CRT electrode was not specifically designed to ensure sufficient LV endocardial penetration of the 3.6 mm tines to capture septal Purkinje tissue. However, whilst LBBAP from an RV approach requires approximately 11–18 mm of septal penetration, it is likely that only superficial penetration is needed from an LV approach, given the close proximity of the LBB to the endocardial surface (Vijayaraman et al., 2019; Elliott et al., 2020; Elliott et al., 2021). demonstrated using electrocardiographic imaging (ECGi) that temporary LV endocardial pacing at the LBP site resulted in improved dyssynchrony metrics compared to conventional CRT, and similar results to HBP and BiV endocardial pacing from the RV and lateral LV. Epicardial EAMs revealed that both HBP and LBBAP result in early septal activation followed by rapid LV activation, consistent with His-Purkinje network recruitment. These EAM studies will need to be replicated following permanent leadless LV pacing targeting the LBP, along with acute haemodynamic testing and follow-up data, in order to determine the efficacy of this technique.

## Discussion: Limitations, Unanswered Questions and Future Directions of Leadless LBBAP

Leadless LBBAP is an exciting new technology which combines the fields of CSP and leadless CRT to provide treatment for a challenging and high-risk group of patients with heart failure. In the short term, improved operator experience will promote more widespread uptake. Refinements to this novel implantation technique will improve safety, success rate, streamline workflow, and reduce parameters such as procedure duration and fluoroscopy time.

Questions remain regarding several areas of leadless LBBAP related to efficacy and patient selection, which will determine its eventual utility amongst all available CRT modalities. Initially, EAM studies are needed to determine whether LV septal WiSE-CRT implantation targeting the LBP achieves conduction system capture, as is postulated by current limited data (Elliott et al., 2020; Elliott et al., 2021). Such studies would also address pertinent questions in the field of CSP regarding potential dyssynchrony. Several small studies and case series report dyssynchrony with lead-based CSP, although the cause of this remains unclear (Arnold et al., 2021). performed ECGi on 20 patients with conventional HBP devices. They reported that “non-selective” HBP, that is, capture of both Purkinje tissue and the surrounding myocardium, caused basal RV pre-excitation compared to “selective” HBP, that is, pure conduction system capture. However, there was no significant difference in LVAT between selective and non-selective HBP capture. Interestingly, other studies have reported that selective-LBBAP causes dyssynchrony due to delayed RV activation, rather than RV pre-excitation (Strocchi et al., 2020). studied ventricular activation on 24 four-chamber heart meshes in the presence of simulated proximal LBBB, using various pacing modalities. They reported that whilst LVAT was reduced with LBBAP compared to BiV pacing, interventricular dyssynchrony was not, as LBBAP resulted in increased RV activation time. Dyssynchrony improved when the atrioventricular delay (AVD) was optimised to allow native RV conduction. These results are consistent with current *in vivo* data, where acute haemodynamic improvements have been noted with AVD optimisation in both lead-based HBP (Padeletti et al., 2016) and LBBAP (Huang et al., 2020) to allow fusion between the LV paced wavefront and intrinsic RV conduction. Further *in vivo* mechanistic studies are needed to clarify the biventricular activation pattern achieved with leadless LBBAP, whether there is significant dyssynchrony, and whether there are adequate programming algorithms to overcome this effectively in the WiSE-CRT setting.

Another area of interest is which patients are most likely to benefit from leadless LBBAP. The CRT population is heterogenous, comprising various conduction disease phenotypes and cardiomyopathy aetiologies, often with complex left-sided electrical activation patterns. How LBBAP performs in the presence of these factors, both in the lead-based and leadless settings, remains unclear.

It is well established from studies of conventional CRT that pacing in areas of scar is predictive of poor response (Bleeker et al., 2006; Chalil et al., 2007; Leyva et al., 2011; Wong et al., 2013). This phenomenon is also seen during LV endocardial pacing (Behar et al., 2016). With regards to the effect of scar on LBBAP, simulated data from four-chamber heart meshes has suggested that in the presence of septal scar, standard BiV pacing is superior to CSP, whilst in the presence of lateral LV scar, CSP outperforms BiV pacing (Strocchi et al., 2021a). As yet, this has not been tested *in vivo*. In part, this is because the presence of septal scar creates technical difficulty in placing a pacing lead to the LBBA with the standard technique of deep interventricular septal deployment (Ali et al., 2021). Leadless LBBAP overcomes this issue, and studies are warranted to determine the performance of LV endocardial CSP in the presence of various scar patterns, especially as studies thus far have only examined myocardial pacing, rather than His-Purkinje capture.

A related area is the effect of His-Purkinje and myocardial conduction velocity on outcomes of CSP. Upadhyay et al. (2019) reported that in patients with LBBB caused by proximal His-Purkinje block, HBP effectively overcomes this, resulting in a narrowing of QRSd in 85% of such patients. However, in their study, patients with “intact Purkinje activation”, that is, where dyssynchrony is caused by distal Purkinje slow conduction velocity or intramyocardial myocardial conduction delay, exhibited no improvement with HBP. These results are consistent with in-silico findings, which showed that normal His-Purkinje velocity favoured HBP, whereas slow velocity favoured standard BiV pacing (Supplementary Appendix Figure S3) (Strocchi et al., 2021b). Conduction velocity is a metric which is generally not measured in routine clinical practice, however, this can be easily incorporated into the workflow of an LV endocardial septal implant, where a decapolar electrophysiology catheter is used to locate Purkinje potentials as the target site for electrode deployment. If *in vivo* studies corroborate the in-silico data, it is possible that measurement of conduction velocity could be used peri-procedurally within standard WiSE-CRT implantation workflow to guide a decision on whether a LBBAP or lateral wall implant is performed.

With regards to long-term directions for leadless LBBAP, its place in the field of CRT will ultimately be determined by future clinical trials testing its safety and efficacy compared to conventional CRT and lateral wall endocardial pacing. Further improvements in the leadless technology, such as longer battery life and the ability to LV pace without an associated co-implant, are also in progress. If these developments come to fruition, there is potential for the expansion of indications for leadless LBBAP to become a first-line CRT modality in selected patients.

## Data Availability

The original contributions presented in the study are included in the article/Supplementary Material, further inquiries can be directed to the corresponding author.

## References

[B1] Ali N. Arnold A.D. Miyazawa A.A. Keene D. Peters N. S. (2021). Septal Late Gadolinium Enhancement on Cardiac MRI Predicts Failure to Achieve Left Bundle Pacing. Eur. Heart J. - Cardiovasc. Imaging 22, 92. 10.1093/ehjci/jeab090.021 31764982

[B2] Arnold A. D. Shun-Shin M. J. Ali N. Keene D. Howard J. P. Chow J.-J. (2021). Left Ventricular Activation Time and Pattern Are Preserved with Both Selective and Nonselective His Bundle Pacing. Heart Rhythm O2 2, 439–445. 10.1016/j.hroo.2021.08.001 34667958 PMC8505200

[B3] Auricchio A. Delnoy P.-P. Butter C. Brachmann J. Van Erven L. Spitzer S. (2014). Feasibility, Safety, and Short-Term Outcome of Leadless Ultrasound-Based Endocardial Left Ventricular Resynchronization in Heart Failure Patients: Results of the Wireless Stimulation Endocardially for CRT (WiSE-CRT) Study. Europace 16, 681–688. 10.1093/europace/eut435 24497573

[B4] Behar J. M. Jackson T. Hyde E. Claridge S. Gill J. Bostock J. (2016). Optimized Left Ventricular Endocardial Stimulation Is Superior to Optimized Epicardial Stimulation in Ischemic Patients with Poor Response to Cardiac Resynchronization Therapy: A Combined Magnetic Resonance Imaging, Electroanatomic Contact Mapping, and Hemodynamic Study to Target Endocardial Lead Placement. JACC Clin. Electrophysiol. 2, 799–809. 10.1016/j.jacep.2016.04.006 28066827 PMC5196018

[B5] Bernard M. L. (2016). Pacing without Wires: Leadless Cardiac Pacing. Ochsner. J. 16, 238. 27660571 PMC5024804

[B6] Bleeker G. B. Schalij M. J. van der Wall E. E. Bax J. J. (2006). Postero-Lateral Scar Tissue Resulting in Non-response to Cardiac Resynchronization Therapy. J. Cardiovasc Electrophysiol. 17, 899–901. 10.1111/j.1540-8167.2006.00499.x 16903969

[B7] Bollmann A. Ueberham L. Schuler E. Wiedemann M. Reithmann C. Sause A. (2018). Cardiac Tamponade in Catheter Ablation of Atrial Fibrillation: German-Wide Analysis of 21 141 Procedures in the Helios Atrial Fibrillation Ablation Registry (Safer). Europace 20, 1944–1951. 10.1093/europace/euy131 29982554

[B8] Bordachar P. Grenz N. Jais P. Ritter P. Leclercq C. Morgan J. M. (2012). Left Ventricular Endocardial or Triventricular Pacing to Optimize Cardiac Resynchronization Therapy in a Chronic Canine Model of Ischemic Heart Failure. Am. J. Physiol. Heart Circ. Physiol. 303, H207–H215. 10.1152/ajpheart.01117.2011 22561299

[B9] Carabelli A. Jabeur M. Jacon P. Rinaldi C. A. Leclercq C. Rovaris G. (2021). European Experience with a First Totally Leadless Cardiac Resynchronization Therapy Pacemaker System. Europace 23, 740–747. 10.1093/europace/euaa342 33313789 PMC8139811

[B10] Chalil S. Foley P. W. X. Muyhaldeen S. A. Patel K. C. R. Yousef Z. R. Smith R. E. A. (2007). Late Gadolinium Enhancement-Cardiovascular Magnetic Resonance as a Predictor of Response to Cardiac Resynchronization Therapy in Patients with Ischaemic Cardiomyopathy. Europace 9, 1031–1037. 10.1093/europace/eum133 17933857

[B11] Derval N. Steendijk P. Gula L. J. Deplagne A. Laborderie J. Sacher F. (2010). Optimizing Hemodynamics in Heart Failure Patients by Systematic Screening of Left Ventricular Pacing Sites: the Lateral Left Ventricular Wall and the Coronary Sinus are Rarely the Best Sites. J. Am. Coll. Cardiol. 55, 566–575. 10.1016/j.jacc.2009.08.045 19931364

[B12] Echt D. S. Moore D. Cowan M. Valli V. E. Whitehair J. G. (2010). Chronic Implantation of Leadless Pacing Electrodes in the Left Ventricle of a Goat Model. Heart rhythm. 7, S451–S452. 10.1016/j.hrthm.2010.03.035

[B13] Elliott M. K. Jacon P. Sidhu B. S. Smith L. J. Mehta V. S. Gould J. (2021). Technical Feasibility of Leadless Left Bundle Branch Area Pacing for Cardiac Resynchronization: a Case Series. Eur. Heart J. Case Rep. 5, ytab379. 10.1093/ehjcr/ytab379 34859181 PMC8633604

[B14] Elliott M. K. Mehta V. Sidhu B. S. Niederer S. Rinaldi C. A. (2020). Electrocardiographic Imaging of His Bundle, Left Bundle Branch, Epicardial, and Endocardial Left Ventricular Pacing to Achieve Cardiac Resynchronization Therapy. Hear. Case Rep. 6, 460–463. 10.1016/j.hrcr.2020.04.012 PMC7361176 32695602

[B15] Gamble J. H. P. Herring N. Ginks M. Rajappan K. Bashir Y. Betts T. R. (2016). Procedural Success of Left Ventricular Lead Placement for Cardiac Resynchronization Therapy: A Meta-Analysis. JACC Clin. Electrophysiol. 2, 69–77. 10.1016/j.jacep.2015.08.009 29766856

[B16] Garrigue S. Jaïs P. Espil G. Labeque J.-N. Hocini M. Shah D. C. (2001). Comparison of Chronic Biventricular Pacing between Epicardial and Endocardial Left Ventricular Stimulation Using Doppler Tissue Imaging in Patients with Heart Failure. Am. J. Cardiol. 88, 858–862. 10.1016/s0002-9149(01)01892-6 11676947

[B17] Ginks M. R. Lambiase P. D. Duckett S. G. Bostock J. Chinchapatnam P. Rhode K. (2011). A Simultaneous X-Ray/MRI and Noncontact Mapping Study of the Acute Hemodynamic Effect of Left Ventricular Endocardial and Epicardial Cardiac Resynchronization Therapy in Humans. Circ. Heart Fail. 4, 170–179. 10.1161/circheartfailure.110.958124 21216832

[B18] Glikson M. Nielsen J. C. Kronborg M. B. Michowitz Y. Auricchio A. Barbash I. M. (2021). 2021 ESC Guidelines on Cardiac Pacing and Cardiac Resynchronization Therapy. Eur. Heart J. 42, 3427–3520. 10.1093/eurheartj/ehab364 34586378

[B19] Hua W. Fan X. Li X. Niu H. Gu M. Ning X. (2020). Comparison of Left Bundle Branch and His Bundle Pacing in Bradycardia Patients. JACC Clin. Electrophysiol. 6, 1291–1299. 10.1016/j.jacep.2020.05.008 33092757

[B20] Huang W. Chen X. Su L. Wu S. Xia X. Vijayaraman P. (2019). A Beginner's Guide to Permanent Left Bundle Branch Pacing. Heart rhythm. 16, 1791–1796. 10.1016/j.hrthm.2019.06.016 31233818

[B21] Huang W. Wu S. Vijayaraman P. Su L. Chen X. Cai B. (2020). Cardiac Resynchronization Therapy in Patients with Nonischemic Cardiomyopathy Using Left Bundle Branch Pacing. JACC Clin. Electrophysiol. 6, 849–858. 10.1016/j.jacep.2020.04.011 32703568

[B22] Leyva F. Foley P. W. Chalil S. Ratib K. Smith R. E. Prinzen F. (2011). Cardiac Resynchronization Therapy Guided by Late Gadolinium-Enhancement Cardiovascular Magnetic Resonance. J. Cardiovasc Magn. Reson 13, 29–9. 10.1186/1532-429X-13-29 21668964 PMC3141552

[B23] Long D.-Y. Dong J.-Z. Sang C.-H. Jiang C.-X. Tang R.-B. Yan Q. (2013). Isolated Conduction within the Left His-Purkenje System during Sinus Rhythm and Idiopathic Left Ventricle Tachycardia: Findings From Mapping the Whole Conduction System. Circ Arrhythmia Electrophysiol. 6, 522–527. 10.1161/circep.113.000293 23673906

[B24] McAlister F. A. Ezekowitz J. Hooton N. Vandermeer B. Spooner C. Dryden D. M. (2007). Cardiac Resynchronization Therapy for Patients with Left Ventricular Systolic Dysfunction. JAMA 297, 2502. 10.1001/jama.297.22.2502 17565085

[B25] Mendonca Costa C. Neic A. Kerfoot E. Porter B. Sieniewicz B. Gould J. (2019). Pacing in Proximity to Scar during Cardiac Resynchronization Therapy Increases Local Dispersion of Repolarization and Susceptibility to Ventricular Arrhythmogenesis. Heart rhythm. 16, 1475–1483. 10.1016/j.hrthm.2019.03.027 30930329 PMC6774764

[B26] Morgan J. M. Biffi M. Gellér L. Leclercq C. Ruffa F. Tung S. (2016). ALternate Site Cardiac ResYNChronization (ALSYNC): a Prospective and Multicentre Study of Left Ventricular Endocardial Pacing for Cardiac Resynchronization Therapy. Eur. Heart J. 37, 2118–2127. 10.1093/eurheartj/ehv723 26787437

[B27] Okabe T. Hummel J. D. Bank A. J. Niazi I. K. McGrew F. A. Kindsvater S. (2022). Leadless Left Ventricular Stimulation with WiSE-CRT System - Initial Experience and Results from Phase I of SOLVE-CRT Study (Nonrandomized, Roll-In Phase). Heart rhythm. 19, 22–29. 10.1016/j.hrthm.2021.06.1195 34332966

[B28] Padala S. K. Cabrera J. A. Ellenbogen K. A. (2021). Anatomy of the Cardiac Conduction System. Pacing Clin. Electrophysiol. 44, 15–25. 10.1111/pace.14107 33118629

[B29] Padala S. K. Ellenbogen K. A. (2020). Left Bundle Branch Pacing Is the Best Approach to Physiological Pacing. Heart Rhythm O2 1, 59–67. 10.1016/j.hroo.2020.03.002 34113859 PMC8183895

[B30] Padeletti L. Pieragnoli P. Ricciardi G. Innocenti L. Checchi L. Padeletti M. (2016). Simultaneous His Bundle and Left Ventricular Pacing for Optimal Cardiac Resynchronization Therapy Delivery: Acute Hemodynamic Assessment by Pressure-Volume Loops. Circ. Arrhythm. Electrophysiol. 9, e003793. 10.1161/CIRCEP.115.003793 27162032

[B31] Padeletti L. Pieragnoli P. Ricciardi G. Perrotta L. Grifoni G. Porciani M. C. (2012). Acute Hemodynamic Effect of Left Ventricular Endocardial Pacing in Cardiac Resynchronization Therapy: Assessment by Pressure-Volume Loops. Circ Arrhythmia Electrophysiol. 5, 460–467. 10.1161/circep.111.970277 22589286

[B32] Reddy V. Y. Miller M. A. Neuzil P. Søgaard P. Butter C. (2017). Cardiac Resynchronization Therapy with Wireless Left Ventricular Endocardial Pacing the SELECT-LV Study. J Am Coll Cardiol. 69, 2119. 10.1016/j.jacc.2017.02.059 28449772

[B33] Salden F. C. W. M. Luermans J. G. L. M. Westra S. W. Weijs B. Engels E. B. Heckman L. I. B. (2020). Short-Term Hemodynamic and Electrophysiological Effects of Cardiac Resynchronization by Left Ventricular Septal Pacing. J. Am. Coll. Cardiol. 75, 347–359. 10.1016/j.jacc.2019.11.040 32000945

[B34] Sidhu B. S. Lee A. W. C. Haberland U. Rajani R. Niederer S. Rinaldi C. A. (2020). Combined Computed Tomographic Perfusion and Mechanics with Predicted Activation Pattern Can Successfully Guide Implantation of a Wireless Endocardial Pacing System. Europace 22, 298. 10.1093/europace/euz227 31504436

[B35] Sieniewicz B. J. Gould J. Porter B. Sidhu B. S. Behar J. M. Claridge S. (2018a). Optimal Site Selection and Image Fusion Guidance Technology to Facilitate Cardiac Resynchronization Therapy. Expert. Rev. Med. Devicese. 15, 555–570. 10.1080/17434440.2018.1502084 PMC6178093 30019954

[B36] Sieniewicz B. J. Behar J. M. Gould J. Claridge S. Porter B. Sidhu B. S. (2018b). Guidance for Optimal Site Selection of a Leadless Left Ventricular Endocardial Electrode Improves Acute Hemodynamic Response and Chronic Remodeling. JACC Clin. Electrophysiol. 4, 860–868. 10.1016/j.jacep.2018.03.011 30025684

[B37] Sieniewicz B. J. Betts T. R. James S. Turley A. Butter C. Seifert M. (2020). Real-world Experience of Leadless Left Ventricular Endocardial Cardiac Resynchronization Therapy: A Multicenter International Registry of the WiSE-CRT Pacing System. Heart rhythm. 17, 1291–1297. 10.1016/j.hrthm.2020.03.002 32165181 PMC7397503

[B38] Sieniewicz B. J. Gould J. S. Rimington H. M. Ioannou N. Rinaldi C. A. (2017). Transseptal Delivery of a Leadless Left Ventricular Endocardial Pacing Electrode. JACC Clin. Electrophysiol. 3, 1333–1335. 10.1016/j.jacep.2017.04.020 29759633

[B39] Sohal M. Shetty A. Niederer S. Chen Z. Jackson T. Sammut E. (2014). Delayed Trans-septal Activation Results in Comparable Hemodynamic Effect of Left Ventricular and Biventricular Endocardial Pacing. Circ Arrhythmia Electrophysiol. 7, 251–258. 10.1161/circep.113.001152 24610742

[B40] Strocchi M. Costa C. M. Neic A. Gillette K. Elliott M. K. Gould J. (2021a). B-Po03-023 His Bundle Pacing Achieves Better Ventricular Synchrony Than Biventricular Pacing in Patients with Scar in the Left Ventricular Free Wall. Heart rhythm. 18, S197–S198. 10.1016/j.hrthm.2021.06.499

[B41] Strocchi M. Neic A. Gillette K. Elliott M. K. Gould J. Behar J. M. (2021b). B-Po04-002 His-Purkinje Conduction Slowing Worsens Response to His Bundle Pacing. Heart rhythm. 18, S280. 10.1016/j.hrthm.2021.06.699

[B42] Strocchi M. Lee A. W. C. Neic A. Bouyssier J. Gillette K. Plank G. (2020). His-bundle and Left Bundle Pacing with Optimized Atrioventricular Delay Achieve Superior Electrical Synchrony over Endocardial and Epicardial Pacing in Left Bundle Branch Block Patients. Heart rhythm. 17, 1922–1929. 10.1016/j.hrthm.2020.06.028 32603781

[B43] Upadhyay G. A. Cherian T. Shatz D. Y. Beaser A. D. Aziz Z. Ozcan C. (2019). Intracardiac Delineation of Septal Conduction in Left Bundle-Branch Block Patterns. Circulation 139, 1876–1888. 10.1161/circulationaha.118.038648 30704273

[B44] van Rees J. B. de Bie M. K. Thijssen J. Borleffs C. J. W. Schalij M. J. van Erven L. (2011). Implantation-Related Complications of Implantable Cardioverter-Defibrillators and Cardiac Resynchronization Therapy Devices: A Systematic Review of Randomized Clinical Trials. J. Am. Coll. Cardiol. 58, 995–1000. 10.1016/j.jacc.2011.06.007 21867832

[B45] Vijayaraman P. Subzposh F. A. Naperkowski A. Panikkath R. John K. Mascarenhas V. (2019). Prospective Evaluation of Feasibility and Electrophysiologic and Echocardiographic Characteristics of Left Bundle Branch Area Pacing. Heart rhythm. 16, 1774–1782. 10.1016/j.hrthm.2019.05.011 31136869

[B46] Wijesuriya N. Elliott M. K. Mehta V. Sidhu B. S. (2022). Leadless Left Ventricular Endocardial Pacing for Cardiac Resynchronization Therapy: A Systematic Review and Meta-Analysis. Heart rhythm., S1547. in press. 10.1016/j.hrthm.2022.02.018 35189383

[B47] Wong J. A. Yee R. Stirrat J. Scholl D. Krahn A. D. Gula L. J. (2013). Influence of Pacing Site Characteristics on Response to Cardiac Resynchronization Therapy. Circ. Cardiovasc. Imaging 6, 542–550. 10.1161/circimaging.111.000146 23741053

[B48] Wouters P. C. Vernooy K. Cramer M. J. Prinzen F. W. Meine M. (2021). Optimizing Lead Placement for Pacing in Dyssynchronous Heart Failure: The Patient in the Lead. Heart rhythm. 18, 1024–1032. 10.1016/j.hrthm.2021.02.011 33601035

[B49] Young J. B. (2003). Combined Cardiac Resynchronization and Implantable Cardioversion Defibrillation in Advanced Chronic Heart Failure. JAMA 289, 2685. 10.1001/jama.289.20.2685 12771115

