# Supplementary figures and images for: Leadless Left Bundle Branch Area Pacing in Cardiac Resynchronisation Therapy: Advances, Challenges and Future Directions
Source: Front Physiol. 2022 Jun 6;13:898866. doi: 10.3389/fphys.2022.898866 (PMC9208327; doi:10.3389/fphys.2022.898866)

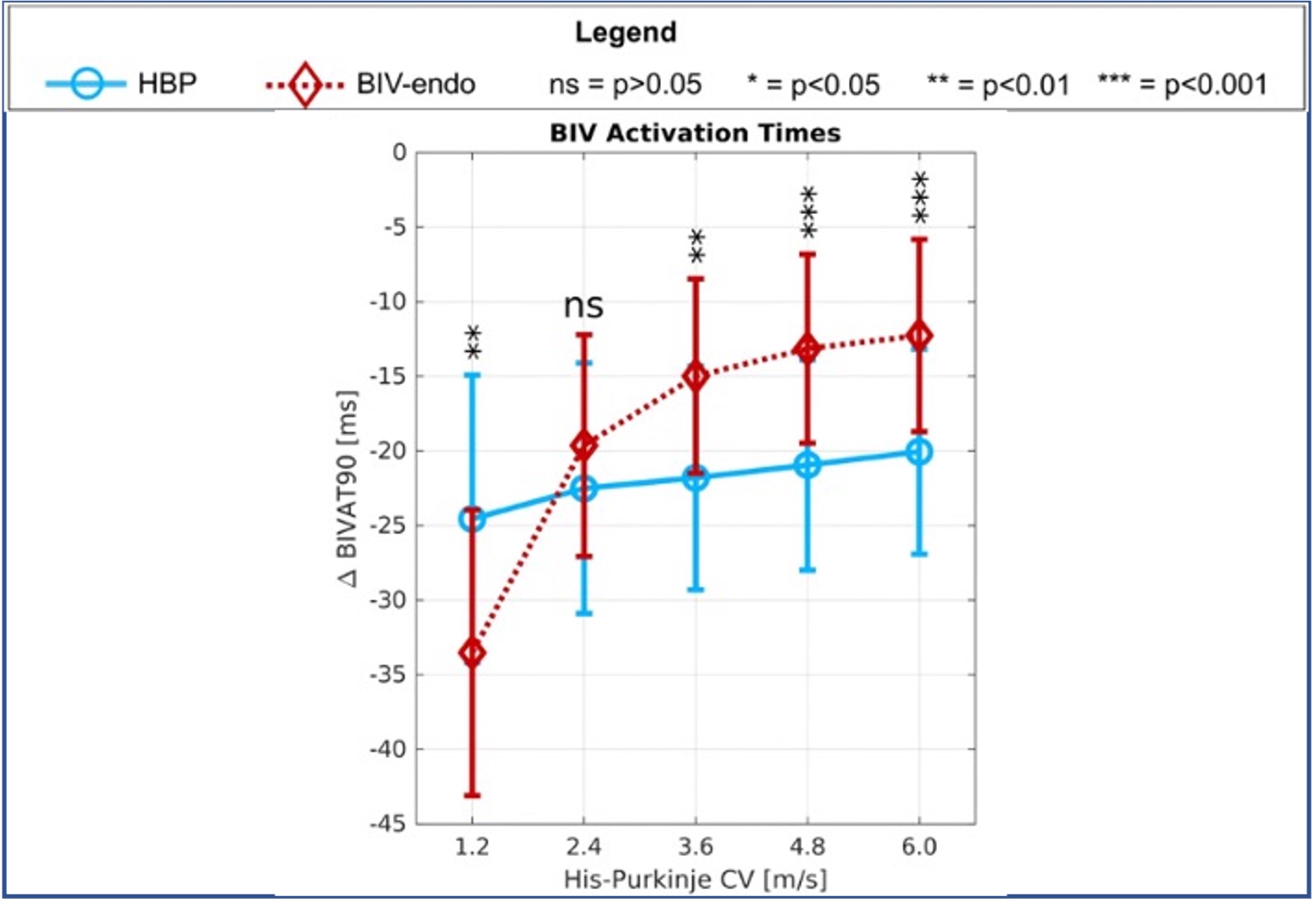

Supplement: Supplementary file 1 [file Image3.jpeg]

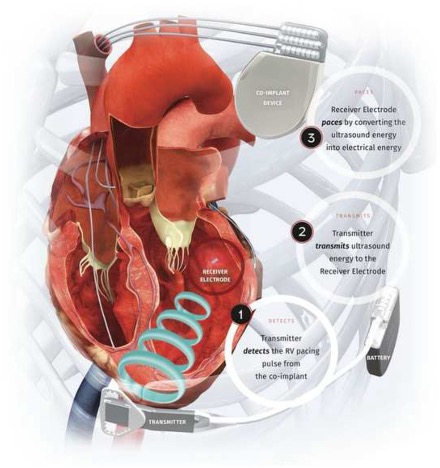

Supplement: Supplementary file 2 [file Image1.jpeg]

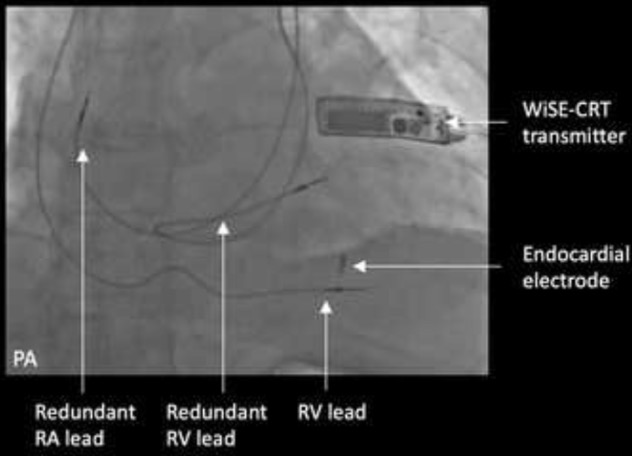

Supplement: Supplementary file 3 [file Image2.jpeg]
